# China and the WHO pandemic treaty: a dive into stance, underpinnings, and implications

**DOI:** 10.3389/fpubh.2024.1335751

**Published:** 2024-01-30

**Authors:** Ying Huang, Shisong Jiang, Emmanuel Kumah

**Affiliations:** ^1^Yangtze Normal University, Chongqing, China; ^2^School of Law, Chongqing University, Chongqing, China; ^3^Department of Health Administration and Education, Faculty of Science Education, University of Education, Winneba, Ghana

**Keywords:** pandemic treaty, China, global health governance, COVID-19, developing countries

## Abstract

The COVID-19 pandemic exposed gaps in global health governance, catalyzing proposals for a new WHO pandemic treaty. This paper investigates China’s stance on the treaty, recognizing it as reflective of many developing countries’ concerns, through a qualitative analysis of its interventions during the treaty’s drafting and negotiations and an examination of historical and geopolitical factors. Findings reveal China’s emphasis on respecting state sovereignty, differentiated obligations for developing nations, preventing stigma, and concrete capacity building—concerns shared across the Global South. Its posture balances pragmatism and principle, reflecting differentiated responsibilities as a major power and developing country along with philosophical divergences from Western legal thinking. While endorsing global cooperation, China insists on voluntary terms without impinging on policy space. Implications suggest that accommodating China’s concerns about invasive compliance mechanisms and inequitable burdens through flexible provisions can shape the treaty’s acceptance and architecture. Creative solutions reconciling sovereignty and collective action combined with concrete equity measures and depoliticized cooperation will determine the treaty’s success. China’s major role indicates its endorsement, representative of the Global South’s voice, is essential for an impactful pandemic treaty and reformed global health governance.

## 1 Introduction

The COVID-19 pandemic, which brought the world to an unprecedented standstill, unveiled substantial gaps in global health governance architecture (1–4). This prompted global leaders to consider innovative legal instruments to manage future pandemics more effectively (5, 6). Amidst this backdrop, the proposition of a pandemic treaty surfaced as a beacon of hope (7). Envisioned to harmonize international efforts against future health crises, its origins can be traced back to the initiative of Charles Michel, the President of the European Council, in November 2020 (8). This proposition soon gained traction, culminating in the World Health Organization (WHO) positioning itself as a conduit for the treaty’s realization (9). By March 2021, a coalition of world leaders rallied behind the cause, advocating for a pandemic treaty under the aegis of the WHO (6). To transform this vision into reality, the World Health Assembly (WHA), in December 2021, initiated the Intergovernmental Negotiating Body (INB), tasking it with the drafting and negotiation of a WHO instrument on pandemic prevention, preparedness, and response (10).

Embarking on this mission, the INB has convened multiple times since late 2021, shaping the contours of the proposed treaty (11). This iterative process has witnessed the creation of the Conceptual Zero Draft (12), the Zero Draft (13), and the Bureau’s text (14). With an aim to deliver its final proposed text to the WHA in May 2024 (15), the INB is actively seeking inputs and navigating complex negotiations (16). Yet, as the INB set forth to shape the treaty’s blueprint, it became evident that the road to a universally accepted treaty was riddled with divergences (17–20). From the very inception of the treaty’s idea to its meticulous drafting, nations grappled with a spectrum of views (21–23), reflecting the intricacies of their national interests, economic stakes, and political aspirations (24–30). These differences have sparked debates around pivotal issues, such as the authority vested in the WHO, equitable access to medical countermeasures, and the fiscal viability of health systems (21).

Amidst this global discourse, China’s stance emerges as pivotal yet underdiagnosed. Contrary to its increasingly assertive role in global health governance (31–33) and international law (34–37), China was conspicuously absent from the pioneering group endorsing the treaty (6, 38). A statement from China’s Ministry of Foreign Affairs ahead of a vote at the WHA on whether to establish a binding treaty or convention articulated China’s position (39). The spokesperson of the Ministry elucidated that “China has always been committed to pandemic preparedness and response and is open to any effort and measure that can help strengthen global solidarity and coordinate responses to future pandemics. We stand ready to maintain communication and coordination with all parties on formulating a pandemic treaty. We hope that the relevant process will move forward within the framework of the UN and the WHO to ensure universal participation of all member states, avoid politicization and stigmatization, and make sure the process will not be used as a tool.” (39) Such a statement that stopped short of an overt endorsement of the treaty, combined with minimal coverage of the ensuing INB’s endeavors in China’s official media, paints a picture of caution. This discernible restraint raises a compelling question: What is China’s stance on the proposed pandemic treaty, and what underlying factors and considerations shape this position?

To address this question, this paper employs a dual analytical approach. The first prong utilizes a rigorous qualitative methodology to examine China’s expressed perspectives from primary data of the INB meetings. An extensive review was conducted of publicly accessible webcasts for all INB sessions to date (as of October 2023). Within these videos, every statement made by Chinese delegates was identified and transcribed verbatim initially in Chinese. To enhance accuracy, the Chinese transcripts were then translated into English and cross-validated against official English interpretations. The resulting textual corpus was compiled into a dataset of China’s interventions across the INB negotiations.

A systematic qualitative coding process was applied to analyze this dataset (40). An initial round of open, inductive coding extracted an extensive set of codes capturing key aspects of China’s discourse. Axial coding then categorized these codes into salient thematic clusters based on semantic relationships. Main themes included sovereignty, global cooperation, compliance, information sharing, and more. Finally, a deductive analysis utilized the finalized coding scheme to elucidate China’s views within each theme systematically. The second analytical prong examines the historical and geopolitical context underlying China’s stance. The analysis delves into China’s past global health engagements, legal philosophy, and prevailing geopolitics as crucial touchpoints to discern strategic motivations. Together, the two prongs synthesize China’s expressed views with historical-geopolitical factors to offer a comprehensive perspective (41).

Accordingly, the ensuing sections unfold as follows: Section 2 presents a thematic analysis of China’s INB interventions, drawing on the qualitative data to elucidate its expressed views. Section 3 interprets the motivations and considerations influencing its stance based on historical engagements, legal approach, and geopolitical context. Section 4 discusses the implications of China’s position and provides recommendations for accommodating its concerns in the treaty framework and broader global governance. Finally, the conclusion synthesizes key points and reflections to offer a comprehensive understanding of the multiple factors molding China’s posture on the proposed pandemic treaty. It is important to clarify that this paper is not intended to support or oppose China’s posture on the pandemic treaty. Instead, it is to offer critical insights and principles that could inform the current and future negotiations and diplomacy.

## 2 Deciphering the dialogue: China’s expressed views in the pandemic treaty negotiations

The INB meetings represent a pivotal forum where nations directly voice their perspectives, concerns, and recommendations on the contours and contents of the treaty (21). China’s interventions (**Table** **1**) in these discussions offer a unique window into its current thinking and priorities. While not definitive, they provide tangible insights into its stance. Bearing this in mind, an examination of China’s expressed views across the INB meetings reveals several salient themes that illuminate its position.

**Table 1:** Overview of China’s interventions on the proposed WHO pandemic treaty.

Content area	China’s position/proposal	Examples of original text
Legal nature and basis of the treaty	Clarify binding vs. non-binding nature; Determine if under Article 19 or 21 of the WHO Constitution; Define early in the treaty development process; Define relationship with IHR revision process.	“Firstly, whether the treaty is to be developed under Article 19 or 21 of the Constitution, the nature of the international instrument should be clarified as soon as possible.” (INB2 Day 1)
Scope and relationship with IHR	Delineate scope between treaty and IHR; Avoid overlap and duplication; WHO Secretariat to map issues between treaty and IHR.	“It’s important to clarify the issues that the international instrument is intended to focus on..suggests that the Bureau and the Secretariat conduct a preliminary screening and analysis of the elements proposal proposed by Member States based on the relevant work done in the previous period, and it’s not appropriate to repeat the discussions on the INB mechanism for issues that can be resolved through other means.” (INB2 Day 1)
National sovereignty	Respect state sovereignty over health measures; Caution against invasive external oversight; Propose softened language like “should duly consider interests of others.”	“Member States have the right to manage and regulate their public health measures.” (INB2 Day 1) “...we suggest modifying “provided that activities within their jurisdiction or control do not cause damage to other States and their peoples” to “should duly consider the interests of other countries and their people.”” (INB3 Day 2)
Differentiated obligations	Strengthen capacities of developing countries; Ensure equity in countermeasures, tech transfer, and financing; Developed nations provide more support; Caution against unrealistic obligations on developing countries.	“China believes that the scope of equity should be broader in addition to equitable access to prevention and control tools. It is also important to improve the capacity of developing countries to prevent, protect against, and respond to health emergencies.” (INB2 Day 2)
Information sharing	Support sharing pandemic info and pathogens; Align with CBD, Nagoya Protocol on benefit sharing; Address stigmatization.	“Information and pathogen sharing are important for Member States to better understand the situation, conduct risk assessments, and respond accordingly.” (INB2 Day 1) “Align with CBD, Nagoya Protocol on equitable benefit sharing” (INB3 Day 2)
Global cooperation	Support cooperation based on state consent and sovereignty; Caution against invasive mechanisms without consent; Favored flexibility like allowing reservations.	“Enhancing coordination, collaboration, and cooperation between countries is crucial for pandemic control.” (INB3 Day 2) “…we suggest that the treaty should maintain flexibility, allowing contracting parties to make reservations to attract more countries to sign.” (INB3 Day 2)
Compliance mechanisms	Design oversight respecting state sovereignty; Consider capacities of developing countries; Avoid invasive compliance measures.	“Compliance mechanisms and accountability measures are core concerns for all parties. From a chronological perspective, the relevant mechanisms and measures should be clarified first, after which countries can decide whether to join the treaty. These mechanisms and measures should respect national sovereignty..” (INB3 Day 2)

### 2.1 Clarifying legal scope and status

A core theme in China’s interventions is urging clarity on the legal nature and scope of the proposed pandemic treaty early in the process. In multiple INB meetings, China has stressed the need to decide whether the treaty will be legally binding or a non-binding cooperation framework. For instance, in INB2, the Chinese delegation stated, “the nature of the international instrument should be clarified as soon as possible. Whether it should be a framework convention containing only guidance in principle, or a regulation with a detailed and implementable actions” (INB2 Day 1).

The question of binding status has legal implications regarding which WHO Constitution provision the treaty could be adopted under - Article 19 for conventions or Article 21 for regulations. China has repeatedly called for clarifying this, saying in INB2 “whether the treaty is to be developed under Article 19 or 21 of the Constitution, the nature of the international instrument should be clarified as soon as possible” (INB2 Day 1).

Beyond binding status, China has insisted on delineating the pandemic treaty’s scope and relationship with the parallel International Health Regulations (IHR) amendment process. It wants to avoid duplication and inconsistencies between the two instruments. In INB4, China requested effectiveness in avoiding “overlaps and, especially, conflicts” with the IHR revisions (INB4). This emphasis reflects China’s overarching concern about precisely defining the treaty’s legal nature and scope early on before specifics are negotiated.

### 2.2 Respect for national sovereignty

The second salient theme is China’s unyielding advocacy for firmly cementing state sovereignty and authority over public health governance in the treaty. In INB2, China stated, “Member States have the right to manage and regulate their public health measures” (INB2 Day 1). In INB3, it called for treaty formulations “respecting sovereignty” of states (INB3 Day 1).

China has repeatedly cautioned against external oversight mechanisms that could infringe on national sovereignty. On compliance, it noted in INB3 that “relevant mechanisms and measures should respect national sovereignty” (INB3 Day 2). Regarding investigations, it is remarked in INB5 that “when WHO decides to conduct investigations in affected areas, it must do so respecting national sovereignty, based on national needs, and with the consent of the country” (INB5).

Furthermore, China has proposed modifications to language that could potentially impose expansive obligations on states. In INB3, it suggested changing the text requiring states to not “cause damage” to others to the softened phrase “should duly consider the interests of other countries” (INB3 Day 2). These statements demonstrate China’s insistence on firmly anchoring state consent and sovereignty in the treaty rather than ceding discretion.

### 2.3 Differentiated obligations

The data also reveals that China consistently advocates differentiated obligations calibrated to countries’ economic development levels and capacities. In INB2, China called for “improving the capacity of developing countries” for pandemic preparedness and response (INB2 Day 1). It has stressed that developed countries should furnish more support to developing nations. In INB5, China endorsed “concrete mechanisms to build developing countries’ capacities” such as technology transfer and financing (INB5).

China has cautioned against unrealistic obligations on poorer countries. In INB3, it noted oversight mechanisms should account for “the pronounced capacity issues of developing countries” (INB3 Day 2). This emphasis on differentiated commitments tailored to countries’ capacities reflects China’s overarching concern about equity and avoiding undue burden on the developing world.

### 2.4 Information sharing with protection against stigma

China has consistently advocated for sharing pandemic outbreak information and pathogen samples to enable risk assessments and vaccine development. However, it has also cautioned against stigmatizing countries that report novel pathogens, which can deter transparency. In INB2, China remarked that “information and pathogen sharing are important for Member States to better understand the situation, conduct risk assessments, and respond accordingly” but also raised “tackling stigmatization” of reporting countries as a key principle (INB2 Day 1).

China has also underscored aligning regulations on sharing biological materials with the Convention on Biological Diversity (CBD) and Nagoya Protocol principles of equitable benefit sharing. In INB3, it stated, “sharing of pathogens and genetic sequences should be in line with domestic and international legal frameworks” like CBD (INB3 Day 2). Overall, China supports global outbreak information sharing with protections against stigma and in line with other instruments.

### 2.5 Global cooperation on flexible terms

The data reveals that China is endorsing global coordination and collaboration against pandemics, but within the bounds of national sovereignty and based on voluntary cooperation rather than invasive legally binding mechanisms. In INB5, China remarked that “enhancing coordination, collaboration, and cooperation between countries is crucial for pandemic control” but emphasized that WHO investigations require “consent of the country” (INB5).

Regarding compliance, China noted in INB3 that oversight mechanisms should “emphasize state-led and state-consented principles, avoiding invasive mechanisms” (INB3 Day 2). In INB3, it favored the flexibility of “allowing contracting parties to make reservations” to widen participation (INB3 Day 3). These statements reflect China’s overarching preference for non-binding global cooperation on flexible terms over strong enforcement provisions that restrict policy space.

### 2.6 Accounting for limited capacities

A final theme is China urging compliance and accountability mechanisms in the treaty that account for limited developing country capacities. In INB3, it stated that oversight mechanisms should “take into account the pronounced capacity issues of developing countries” (INB3 Day 2). It has consistently cautioned against unduly invasive external enforcement measures, noting in INB5 the need to avoid “invasive compliance mechanisms” (INB5).

These concerns reflect China’s insistence that the treaty reasonably accommodates development gaps and constraints on poorer countries’ capacities rather than taking a punitive approach towards non-compliance. It worries about punitive enforcement against developing states.

## 3 Underlying currents: unpacking the reasons behind China’s stance on the proposed pandemic treaty

China’s perspective on the proposed pandemic treaty reflects a confluence of historical experiences, philosophical orientations, economic capacities, and geopolitical considerations that shape its stance. An examination of these underlying factors provides insights into China’s intricate balancing act between pragmatism and principle as it navigates the treaty negotiations.

### 3.1 Diverging legal philosophies and priorities

China’s legal and philosophical traditions diverge markedly from Western legal thinking, stemming from its distinct historical and cultural lineage (37, 42, 43). Central to this is China’s emphasis on state sovereignty and freedom from external constraints that could impinge on its policy maneuverability (44, 45). China prioritizes its sovereign discretion over binding international legal obligations that could limit its flexibility (46, 47). This foundational principle manifests in its posture on the pandemic treaty, evident in its insistence on voluntary cooperation instead of stringent, legally binding enforcement mechanisms.

China’s general approach to international agreements and engagements reveals its preference for political commitments that preserve latitude over rigid treaties. Its deals under the Belt and Road Initiative, for example, frequently adopt informal Memorandums of Understanding rather than strict, binding contracts (47). Even its “mask diplomacy” during the early pandemic relied predominantly on informal arrangements rather than formal treaties (48). This modus operandi underscores China’s cautious stance on the pandemic treaty, as it seeks to first assess the implications for its sovereignty and policy space before acceding to binding provisions.

To a large extent, China’s legal philosophy is steeped in a cultural tradition that emphasizes social harmony and moral suasion over rigid laws and harsh punishments (49). It shares more similarities with Confucian relational models than Western rule of law concepts (50). China puts greater stock in building interpersonal relationships, mutual understanding, and voluntary cooperation to resolve disputes (51). This contrasts with the Western proclivity for impersonal institutions, codified rules, and coercive enforcement mechanisms (52). These divergent legal mentalities could shape China’s insistence on voluntary participation over punitive measures in the pandemic treaty.

### 3.2 Different capabilities and responsibilities

China’s stance may also stem from its self-perception as a developing country with differentiated responsibilities compared to Western powers (53). While acknowledging its major power status, China remains conscious of its domestic development challenges (54). Vast disparities persist, for instance, between its advanced urban centers and impoverished rural hinterlands (55). China consistently advocates for obligations commensurate with respective capabilities in global agreements, mirroring these internal imbalances (56).

Despite making economic progress, China’s financial capacity for foreign aid remains modest compared to some developed economies. Its pledge of around $3 billion over three years for pandemic response and recovery in developing nations (57) pales beside the scale of relief enacted by wealthier countries. The United States alone has passed laws dedicating approximately $4.5 trillion towards its domestic and international COVID-19 response as of early 2023, with $4.2 trillion already expended (58). With a GDP *per capita* of over $12,000, China ranks 63rd globally (59), a position that still lags behind most developed economies. Mindful of these fiscal and development constraints, China continues to exhibit wariness about over-committing to expansive global accords that could impose obligations exceeding its current material capacities (60). When engaging with proposed international agreements like the pandemic treaty, it seems unsurprised that China may want to avoid being bound to treaty commitments that surpass its financial and institutional abilities to fulfill at the present moment.

In many international organizations, including the World Trade Organization (WTO), China continues to categorize itself as a developing country (61). This self-identification significantly shapes its posture on global agreements, driving its pursuit for differentiated responsibilities rather than equal obligations (62). This stance is also evident in its advocacy for carve-outs and graduated expectations in the pandemic treaty. While its economic and geopolitical prominence is rising steadily (63), China remains conscious of its developmental shortcomings and is unlikely to relinquish the flexibilities afforded to the developing world readily (64).

### 3.3 Unfair stigmatization and politicization

The politicization and stigmatization surrounding the COVID-19 pandemic (65, 66) may also have impacted China’s treaty approach. China feels it has been unfairly scapegoated for the outbreak (67). Accusations of cover-ups and negligence, along with the label “China virus,” have bred resentment. Such rhetoric has fueled racist attacks against Chinese people overseas (68). Calls for politically motivated probes into the virus’s origins further heighten distrust and politicization from China’s vantage point (69).

Consequently, China’s emphasis on avoiding politicization, stigmatization, and instrumentalization in the pandemic treaty text reflects these grievances (39). It seeks to preempt any attempts to single out or unfairly assign blame on China through the treaty’s provisions. Having faced the brunt of politicized narratives, China grows wary of clauses that could engender similar mistreatment.

China believes the pandemic rhetoric reveals the West’s plot to undermine its power and prestige. Terms like “China virus” or “Wuhan virus” are seen as deliberate ploys to disparage China and shift blame (69). Demands for compensation from litigious Western politicians [e.g., (70)] exacerbated these fears of directed vilification. The initial push for the treaty by Western powers rekindled suspicions of an orchestrated effort to pin responsibility on China. Its defensive stance stems from apprehensions that the treaty could formalize such recrimination.

### 3.4 Western-centric global governance

Historically, China has felt sidelined within global governance architectures centered around Western interests. It perceives many existing regimes as Western-dominated, catering to Northern agendas while disregarding Southern priorities (71). During negotiations for the IHR, for instance, language barriers, technical complexities, and other factors marginalized China (72) as well as other Asian countries (73). Its interests were not adequately represented in a process steered by Western powers (73).

The initial pandemic treaty spearheaded by the EU and G7 revived these concerns about Western-centric governance (23, 29). It is thus reasonable to believe that China fears the treaty could impose inequitable expectations on developing nations without addressing their constraints. Correspondingly, its demands for capacity building, technology transfer, and financial support in its interventions in the INB meetings are no more than a reflection of the anxieties about Northern-driven formulations ignoring Southern needs. In this sense, these argued suspicions of China could be perceived as stemming from a sense of exclusion from setting global rules that it nonetheless must abide by. After all, it is a fact that multilateral institutions like the World Bank and WTO were molded by Western powers with minimal Chinese input (52). As China’s power expands, therefore, it increasingly chafes under systems designed without its participation (36, 74); and the pandemic treaty seems to offer an avenue to rectify perceived imbalances in global governance.

## 4 Implications and ways forward

The analysis presented in Section 3 of this paper elucidates the underlying reasons for China’s stance on the pandemic treaty—a stance that, to a considerable extent, mirrors the experiences and concerns of developing countries as well (18, 23, 28, 29, 75). After all, China’s stance does reflect perspectives shared by many developing nations based on the regional consultations of INB’s process of developing a pandemic treaty (76). For instance, Nigeria’s push for enforceable equity [(76): Annex p.2], Brazil’s sovereignty safeguarding [(76): Annex p.3], and India’s preference for national autonomy over global mandates [(76): Annex p.4] collectively resonate with China’s approach. This confluence of interests, coupled with a shared desire for technological collaboration voiced by the Americas [(76): Annex p.3], underscores a common wariness among these developing countries about the treaty’s potential for political exploitation and stigmatization. In effect, these shared perspectives are rooted in historical challenges, socio-economic disparities, and a desire for more equitable participation in global health governance (77). As such, the implications of China’s position outlined in this section are not unique to China alone but resonate with a collective voice emanating from the Global South.

This commonality will inevitably influence the treaty’s architecture, acceptance, and subsequent implementation. The Bureau’s text, with its various options for certain provisions, reflects the ongoing divides between state discretion and global cooperation. These divergences echo China’s emphasis on state sovereignty and differentiated responsibilities, akin to the concerns of other developing nations over stigmatization and Western-centric global governance models. The intricate balance between sovereignty principles and collective obligations, as highlighted in the Bureau’s Article 17 (14), emerges as a pivotal tension in the ongoing negotiations. China’s potential leaning towards options that prioritize its guiding principles might dictate the very design and structure of the treaty, setting a precedent for other developing countries. This suggests a possible shift toward a framework that is more cooperative than coercive, emphasizing mutual assistance, capacity-building, and technology transfer as underscored in Article 11 (14).

Furthermore, China’s significant influence, especially among nations of the Global South, could play a determining role in the treaty’s acceptance and ratification. Its endorsements, or lack thereof, combined with its advocacy for flexibility, could shape the trajectory of global acceptance. As such, it is essential that the treaty incorporates provisions like Article 6 (14), which promotes preparedness, readiness, and resilience, aligning with the needs and capabilities of developing nations.

Looking ahead, the broader implications of China’s stance extend beyond the treaty’s ratification. Its approach, if integrated into the treaty’s fabric, could redefine the contours of global health governance. While this could usher in a more collaborative era, emphasizing capacity-building and technology transfer, there are also concerns. China’s inherent wariness of external oversight might lead to a treaty with reduced emphasis on stringent monitoring and compliance as outlined in Article 8 (14). This balancing act between accommodating China’s concerns and ensuring global adherence to commitments will be central to the treaty’s effectiveness.

With these overarching considerations in mind, the following delve deeper into specific implications and potential recommendations, shedding light on the complex interplay of China’s position, the treaty’s provisions, and the broader landscape of global health governance.

### 4.1 Sovereignty concerns require innovative accommodations

The Bureau’s text reveals clear fault lines around state sovereignty versus external oversight, reflecting China’s apprehensions. Article 8 enables external assessments of national pandemic preparedness through peer reviews (14), which could be perceived as infringing on China’s jurisdiction without its consent. Similarly, Article 22 proposes an Implementation and Compliance Committee to probe non-compliance issues (14), potentially impinging on sovereign authority over governance matters.

Thus, we suggest that creative technical and legal solutions are necessary to assuage these concerns. Peer reviews in Article 8 could be reimagined as voluntary collaborations rather than mandatory assessments. Oversight bodies like Article 22’s committee may adopt non-adversarial procedures and alternative dispute settlement options like inter-state negotiations. Allowing reservations, delayed entry into force, interim targets, or staged commitments could reconcile principles of sovereignty with binding obligations. Advancing collective interests requires accommodating the discretion concerns of China and other developing countries through elective and gradual measures, rather than rigid review mechanisms.

### 4.2 Equity appeals require concrete commitments

China’s calls for differentiated obligations, reflecting the development levels, necessitate substantive capacity-building and technology transfer pledges, as articulated in Article 17 (14). Without these, China might not commit to binding pandemic response requirements that surpass its current capabilities.

Yet the Bureau’s text, particularly Article 3 (7) (14), lacks concrete mechanisms beyond affirmations of equity. We, therefore, recommend that the treaty should incorporate actionable provisions for needs-based flexibility to motivate China and similarly situated countries. This could include staged implementation pathways, reduced reporting burdens, and binding commitments from developed states on financing, technology transfer, local manufacturing, and technical assistance, as suggested in Article 19 (14). Ensuring equitable representation for the Global South in oversight bodies is also critical. Thus, more than aspirational equity, China and other developing countries require tangible provisions to augment their capabilities.

### 4.3 Overcoming skepticism requires genuine multilateralism

China’s skepticism towards Western-dominated agendas poses a risk to unified action. Contentious issues, such as the pandemic’s origins, fuel China’s mistrust of investigations within its borders without explicit consent, as would be required by Article 15 on declaring pandemics (14). To this end, we believe that preventing the politicization of global health matters requires fostering China’s confidence in principled multilateralism.

Additionally, initial advocacy for a treaty by Western actors may reignite China’s apprehensions of Northern dominance cloaked in cooperation. Thus, Article 20’s Conference of Parties (14) must provide the Global South with a greater voice to avoid China’s defensive disengagement. In sum, a genuine multilateralism that accommodates China’s interests is fundamental to collective resolve.

### 4.4 Health security requires corresponding investments

China has consistently highlighted the need to strengthen health systems and improve pandemic preparedness and response capacities, especially in developing countries. However, the current draft treaty’s provisions lack specific commitments or mechanisms to actualize these goals. It is our view that in order to align better with China’s priorities, the treaty should embed concrete capacity-building mechanisms and commitments from the Global North. This includes needs-based financing, staged capacity targets, mandatory technology transfers, and local manufacturing investments, as exemplified by China’s support for Article 11 (14). Moreover, resilience necessitates investments beyond biosecurity. China’s endorsement of the One Health approach [(14): Article 5], addressing societal, environmental, and economic factors, indicates the need for the treaty to commit to upholding labor rights, food security, gender equality, and environmental sustainability.

## 5 Conclusion

This paper has delved into China’s intricate balancing act in navigating the proposed pandemic treaty negotiations. Several key findings emerge through an integrated analysis of China’s expressed views during INB meetings and the historical, philosophical, and geopolitical factors shaping its stance.

Firstly, respect for state sovereignty permeates China’s posture, reflecting its anchoring legal principles and wariness of external constraints. This manifests in its insistence on voluntary cooperation over invasive compliance mechanisms and differentiated obligations attuned to developmental contexts. However, creative technical and legal solutions like elective reviews and interim targets could bridge China’s sovereignty concerns with collective interests.

Secondly, China’s self-assigned identity as a developing country colors its differentiated responsibilities demand. Cognizant of domestic disparities and aid constraints, it rejects uniform obligations and seeks policy space protections. Yet, with rising capacities, it may be compelled to assume more responsibilities befitting its major power status. This requires nuanced obligations reflecting both principles and pragmatism.

Thirdly, inequitable pandemic experiences reinforced China’s suspicions about Western-dominated governance. Coupled with its accumulating influence, China advocates for greater representation and rebalancing. But with divergent worldviews at play, it must also proactively address apprehensions its ascent sparks in the West. Reconciling perspectives will enable legitimate global leadership.

Fourthly, beyond political wrangling, China endorses initiatives that concretely assist developing nations. Its emphasis on needs-based flexibility and capacity building underscores a development-centered outlook. Constructively channeling its aspirations to uplift the Global South into the treaty’s architecture could catalyze progress.

Fifthly, despite tensions, China’s enthusiastic INB participation reveals its recognition of collective interests at stake. But mistrust risks impeding cooperation. Confidence-building through consistent transparency and principled multilateralism is imperative. Moreover, China’s endorsement would imbue the treaty with legitimacy.

In essence, accommodating China’s interests within overarching global health goals is paramount for an impactful pandemic treaty. This requires nuanced obligations, concrete support, representational reforms, depoliticized cooperation, and integrated worldviews. However, limitations remain in this analysis. Firstly, the lack of access to closed INB drafting group discussions inhibits a comprehensive perspective. China’s unpublished interventions on specific provisions remain obscured. Secondly, minimal interventions from other nations prevent comparative analysis. Broader developing country views could illuminate areas of alignment or divergence with China. Thirdly, China’s public statements may diverge from its private negotiating positions, a discrepancy this paper cannot unpack.

Nonetheless, the synthesis of available evidence offers salient insights and principles to guide future negotiations and diplomacy. As debates continue, constructive engagement with China’s complex considerations, rather than rebuke, promises the most tenable path to global health progress. No doubt, additional research accessing confidential negotiations and expanding the analytical aperture to the developing world’s perspectives can further enrich understanding and discernment.

## Data availability statement

The original contributions presented in the study are included in the article/supplementary material, further inquiries can be directed to the corresponding author.

## Author contributions

YH: Conceptualization, Methodology, Project administration, Writing – original draft, Writing – review & editing. SJ: Conceptualization, Data curation, Formal analysis, Funding acquisition, Investigation, Methodology, Resources, Writing – original draft, Writing – review & editing. EK: Conceptualization, Methodology, Validation, Writing – original draft, Writing – review & editing.
